# Transforming customer experience in social robotics through explainable and interpretable artificial intelligence over a decade

**DOI:** 10.3389/frobt.2026.1693379

**Published:** 2026-03-02

**Authors:** Anshu S. Arora, Amit Arora, John R. McIntyre

**Affiliations:** 1 School of Business and Public Administration, University of the District of Columbia, Washington, DC, United States; 2 Scheller College of Business, Georgia Institute of Technology, Atlanta, GA, United States

**Keywords:** customer experience (CX), explainable artificial intelligence (XAI), human-robot interaction (HRI), interpretable AI (IAI), long-term human-robot trust and relationships, social robotics, socio-behavioral interpretability, transparency

## Abstract

Over the past decade, the field of social robotics has witnessed significant advancements in enhancing user experience (UX) and customer experience (CX) through the integration of Explainable Artificial Intelligence (XAI) and Interpretable Artificial Intelligence (IAI). This research presents a review that examines the progress made over a decade (2015–2025) in developing frameworks for social robotics and human-robot interaction (HRI) that prioritize transparency, trust, and user engagement. The journey began with early efforts to equip social robots with internal needs and motivations, forming the basis for understandable self-explanations. As the field progressed, there was a shift towards more user-centered approaches, autonomous social behavior, and self-explanations. By the early 2020s, researchers had begun to focus on the specific applications of XAI and IAI in social robotics. Past studies have shown that explainable and interpretable AI systems in social robots contributed to sustained user engagement and improved CX over extended periods. Currently, by 2025, the field has matured considerably, with researchers developing comprehensive frameworks that seamlessly integrated UX/CX considerations in social robotics with an emphasis on ethical considerations and societal implications. This research highlights how the past decade has seen remarkable progress in enhancing UX/CX in social robotics through XAI and IAI.

## 1 Introduction

“The social robots market size will grow from $7.66 billion in 2025 to $24.7 billion in 2029 at a compound annual growth rate (CAGR) of 34.0%. Major trends in the forecast period include collaborative robots, artificial intelligence (AI) and machine learning (ML), human-robot interaction, ethical AI and data privacy.”

∼ The Business Research Company Report (2025).^1^

Social robots, designed to engage users through emotional and cognitive connections, have transitioned from niche prototypes to essential tools in retail, healthcare, hospitality, service, education, among several industry categories. Social robot is defined as “a physical entity embodied in a complex, dynamic, and social environment sufficiently empowered to behave in a manner conducive to its own goals and those of its community” (Duffy, 2000). Human-Robot Interaction (HRI) can be broadly defined as the study and design of interactive systems involving humans and robots, with an emphasis on optimizing communication, collaboration, and mutual understanding between them (Esposito et al., 2025; Arora et al., 2024a). Social robotics and HRI research literature indicates that acceptance of the social robot is largely influenced by factors such as the robot’s appearance, usability, perceived ease of use, non-invasiveness of the measurement technology, and perceived usefulness (Ciuffreda et al., 2025).

In the evolving field of human-robot interaction (HRI), understanding the distinct yet interconnected roles of user experience (UX) and customer experience (CX) is crucial, especially with the integration of explainable artificial intelligence (XAI) and interpretable AI (IAI) in social robots. User experience (UX) pertains to users’ direct interactions with robots, emphasizing functionality, usability, emotional engagement, and the transparency of AI decision-making processes (Sanneman and Shah, 2020; Seifi et al., 2024; Lisetschko et al., 2023). Conversely, customer experience (CX) encompasses the broader journey of customers over the lifecycle of robot use, including brand perception, enhanced social HRI experiences with the robot, pre- and post-interaction support, and long-term relationship management (Larivière et al., 2025; Lisetschko et al., 2023). The convergence of UX and CX becomes particularly significant in social robots that fulfill both functional and social roles, necessitating clear and understandable interactions to build trust and encourage adoption.

The acceptance of social robots and the resulting CX outcomes are grounded in several theoretical perspectives. The Computers-Are-Social-Actors (CASA) paradigm (Reeves and Nass, 1996) and the Service Robot Acceptance Model (sRAM) (Wirtz et al., 2018; Song and Kim, 2022) explain how humans apply social norms and service expectations to robotic agents. Similarly, Anthropomorphism Theory and intergroup relationship frameworks such as Intergroup Threat Theory (Vanman and Kappas, 2019) and Speciesism (Fiestas Lopez Guido et al., 2024) help interpret how individuals’ social biases and perceived “otherness” of robots shape trust and long-term acceptance (Yogeeswaran et al., 2016; van Doorn et al., 2017).

Explainable AI (XAI) refers to systems that provide *post hoc* explanations for decisions made by complex models, often used to clarify opaque decision-making in black-box algorithms (Arrieta et al., 2020). In contrast, Interpretable AI (IAI) involves models that are inherently understandable by design, such as decision trees or rule-based systems (Miller, 2019). Early implementations, however, faced significant challenges: opaque decision-making processes in robots, termed the “black box dilemma,” eroded user trust, with a significant percentage of customers reporting discomfort interacting with non-transparent systems (Scheutz et al., 2022). This gap underscored the critical need for XAI and IAI, which prioritize transparency in robotic behavior to align with human social norms and expectations (Arnold et al., 2021; Scheutz et al., 2022).

The emergence of Explainable AI (XAI) and Interpretable AI (IAI) has marked a paradigm shift in social robotics, evolving from *post hoc* explanations toward embedding interpretability directly into algorithmic architectures so robots can articulate motivations and decisions in real time (Stange and Kopp, 2020). XAI gained prominence over the last decade with the rise of deep learning models, whose opaque decision-making structures created demand for interpretability. Post hoc techniques emerged to bridge the gap between complex model behavior and human understanding (Vilone and Longo, 2020; Ehsan et al., 2023; Van Der Velden, 2024), while needs-based frameworks allowed robots to trace internal decision trees and provide context-aware justifications, boosting trust (Stange et al., 2022). Socio-behavioral interpretability layers soon became central to human-robot interaction (HRI), enabling emotionally resonant, understandable actions (Schäfer et al., 2024). For example, Furhat robots deliver dialog-based justifications that enhance satisfaction and perceived expressiveness, especially in low-expectation contexts (Yadollahi et al., 2025). While IAI principles predate XAI through symbolic and rule-based reasoning, modern IAI focuses on embedding interpretability into learning algorithms (Bdiwi et al., 2022). However, XAI’s adaptability risks manipulation or overtrust (Leemann, 2024) and misleading explanations (Martens et al., 2025), prompting a growing trend toward hybrid systems that merge both approaches for socially intelligent robots (Quesada et al., 2022).

This research uniquely contributes to existing literature by systematically connecting the evolution of explainable and interpretable AI (XAI and IAI) in social robotics with shifts in both user experience (UX) and customer experience (CX) over the last decade (2015–2025). While prior reviews have separately examined technical progress or behavioral implications, this study integrates these perspectives within a four-era framework, demonstrating how socio-behavioral interpretability has directly shaped customer trust, long-term human-robot relationships, and practical CX design in real-world domains.

## 2 Methodology

The research presents a scoping review that analyzes a decade (2015–2025) of XAI and IAI advancements in social robotics. This scoping review addressed three guiding questions: (1) How have explainable and interpretable AI architectures in social robotics evolved from 2015 to 2025? (2) How are these developments linked to shifts in user experience (UX) and customer experience (CX) in human–robot interaction? (3) What research gaps and future research directions emerge when XAI/IAI, UX, CX, and social robotics are considered together? The scope offers a holistic perspective on how transparency has transformed social robots from UX functional tools into trusted CX social partners (Mindlin et al., 2025).

To maintain conceptual breadth while tracing the evolution of XAI and IAI in social robotics, this article follows a scoping review approach (Arksey and O’Malley, 2005; Levac et al., 2010) rather than a statistical meta-analysis. We searched Scopus, Web of Science, Google Scholar, and leading HRI and social robotics venues (e.g., Frontiers in Robotics and AI, ACM/IEEE HRI, etc.) using combinations of keywords related to social robots, HRI, explainable/interpretable AI, transparency, trust, UX, and CX, and we used backward and forward citation tracing to identify additional studies. Research Literature (e.g., experimental/quantitative HRI or trust studies, qualitative or observational studies, design or conceptual framework papers, and technical or algorithmic papers with UX/CX implications) from 2015 to 2025 were identified through the above-mentioned academic databases. Search terms included combinations of: “social robots,” “human-robot interaction,” “explainable AI,” “interpretable AI,” “transparency,” “trust,” “user experience (UX),” and “customer experience (CX).”

In this scoping review, we included research studies that addressed explainability or interpretability in the context of social robotics or HRI, and reported user- or customer-relevant outcomes such as UX/CX, trust, autonomy, or socio-behavioral insights derived from interactions with social robots. Highly technical papers without relevance to human interaction or experience were excluded. Work that concentrated solely on technical or algorithmic developments without linking explainable or interpretable AI to human interaction, experiential measures, or real-world HRI contexts was not considered.

The purpose of this research is to evaluate the evolution of self-explanatory architectures and their impact on UX and CX, and assess the integration of interpretable systems into social robots, including societal implications. To fulfil this goal, we synthesized conceptual and technological evolution across four eras, and the screening process prioritized thematic relevance over exhaustive coverage, which is consistent with scoping reviews. This scoping structure supports an integrative understanding of how transparency evolved from functional design features to socio-behavioral trust frameworks, aligning the review with conceptual synthesis standards used in XAI and HRI studies, comparable to Naneva et al. (2020).

**FIGURE 1:**
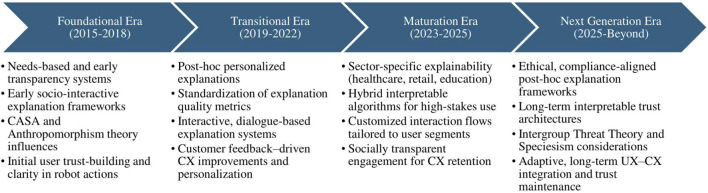
Timeline of key developments in explainable and interpretable AI (XAI and IAI) in social robotics (2015–2025).

Drawing upon the insights generated from the preceding scoping review, we applied an era-based conceptual framework and segmented the decade into four epochs: *Foundational* (2015–2018), *Transitional* (2019–2022), *Maturation* (2023–2025), and *Advanced/Next-Generation* (2025 and beyond), and within each era, we map XAI and IAI foci, theoretical lenses, UX and CX characteristics, and real-world applications using Figure 1 and Table 1. This division highlighted shifts from *post hoc* explanations to inherently interpretable architectures (Nigro et al., 2025). The methodology balanced efficiency with transparency, adhering to Arksey and O’Malley (2005) iterative process, and incorporated the viewpoints of stakeholders (e.g., robotic companies, roboticists, and engineers) (Levac et al., 2010) for generation of social robotics eras highlighting early foundations, transition, maturation, and advancement in social robotics over a decade (2015–2025).

**TABLE 1:** Evolution of explainable AI (XAI) and interpretable AI (IAI) in social robotics (2015–2025 and beyond).

Era	XAI focus	IAI focus	Relevant theories and acceptance drivers	UX (Functional/ Operational)	CX (Social)	Real-Life industry examples
Foundational Era (2015–2018) Early Foundations of Social Robotics	Basic transparency systems, needs-based explanation architectures, early socio-interactive explanation frameworks	Early interpretable rule-based frameworks, transparent decision-making in robotic actions	CASA theory (Reeves and Nass, 1996); Anthropomorphism theory (Yogeeswaran et al., 2016); drivers include customer trust, social presence, perceived usefulness	Basic user transparency and clarity in system actions	Initial trust-building with customers, clear communication of capabilities	NAO Robot in education providing rule-based teaching explanations (Amirova et al., 2021); Pepper Robot in retail explaining product recommendations (Kaptein, 2020; Stange et al., 2022)
Transitional Era (2019–2022) User-Centered/UX Approaches and CX Emergence	Post hoc personalized explanations, metric standardization for explanation quality	Interactive explanation generation embedded into dialogue systems	Service Robot Acceptance Model (sRAM) (Wirtz et al., 2018; Song and Kim, 2022); Technology Acceptance Model extensions; drivers include ease of use, emotional engagement, personalization	Adaptive dialogue design, explanation tailoring based on user profile	Customer feedback-driven social improvements in explanation clarity and personalization	Furhat Robot in customer service with adaptive dialogs (Yadollahi et al., 2025); Pepper Robot in hospitality personalizing greetings (Stange et al., 2022)
Maturation Era (2023–2025) Domain-Specific Applications	Explainability in decision pathways for sector-specific applications	Hybrid interpretable algorithms; inherently interpretable decision models for high-stakes use	Human–Robot Trust Theory (Schaefer, 2016); Social Presence Theory; drivers include transparency, reliability, perceived competence	Customized interaction flows for healthcare, retail, education users	Retention-focused socially transparent engagement in commercial settings	NAO Robot in rehabilitation showing interpretable progress data (Amirova et al., 2021; Cruz-Sandoval et al., 2024); Pepper Robot in healthcare explaining care steps (Gongor and Tutsoy, 2025); Furhat Robot in retail analytics (Recio-Román et al., 2024)
Advanced/Next-Generation Era (2025 – Beyond) Long-Term HRI Relationships and Future Frameworks	Ethical *post hoc* explanation frameworks aligned with regulatory compliance	Long-term interpretable trust frameworks built directly into architectures	Intergroup Threat Theory (Vanman and Kappas, 2019); Speciesism (Fiestas Lopez Guido et al., 2024); Ethical AI Governance Models (Arora et al., 2025a); drivers include cultural adaptability, ethical transparency, long-term trust	Behaviorally adaptive long-term interaction models, diminishing boundaries between UX and CX	Personalized and trust-maintaining engagement over extended relationships	Paro Therapeutic Seal (De Graaf et al., 2021), LOVOT (Joshi, 2023), Unitree G1 Humanoid, Kerfuś Mascot Robot (Recio-Román et al., 2024), ARI SPRING Rehabilitation Robot (Zhou et al., 2024; Mahler, 2024), public-service robots in civic settings (Lim et al., 2021; Babel et al., 2024)

To visually summarize the decade-based segmentation described above, Figure 1 presents a timeline of key developments across the four eras identified in this scoping review. This graphical overview highlights the major milestones, theoretical shifts, interpretability architectures, and UX–CX priorities that characterize each era from 2015 to 2025, showing how social robots move from local, rule-based transparency mechanisms toward socio-behavioral and ethically grounded explainability frameworks. As shown in Figure 1, the four eras reflect a clear progression from foundational transparency mechanisms toward sophisticated, ethically grounded explainability frameworks, which are further detailed in Table 1. Table 1 highlights the evolution and timeline of key milestones of XAI and IAI over a decade (2015–2025 and beyond), emphasizing key developments across four distinct eras: *Foundational* (early foundational robotics architectures), *Transitional* (user-centered UX approaches), *Maturation* (domain-specific applications), and *Advanced/Next-Generation* (long-term HRI relationships and future robotics frameworks) *Eras,* and indicating, for each era, how interpretable mechanisms are instantiated in social robot architectures and interaction modalities, as well as their emerging societal implications.

In synthesizing the selected studies, we explicitly traced how interpretable systems were embedded into concrete social robot architectures (e.g., rule-based dialog and decision modules, hybrid XAI–IAI pipelines that separate decision and explanation layers, needs-based self-explanatory architectures, and LLM–knowledge-graph intention recognition frameworks) and how these designs shaped user-facing behavior in healthcare, retail, education, and public service domains. We also examined societal implications, including long-term trust, anthropomorphism and attachment, cross-cultural acceptance, and alignment with emerging regulatory frameworks such as the EU AI Act (Arora et al., 2025a), particularly for vulnerable populations and public-sector deployments.

## 3 Foundational era: early foundations of social robotics (2015–2018)

### 3.1 Development of internal needs-based architectures

The foundational era of explainable social robotics (2015–2018) saw the emergence of architectures prioritizing *internal needs* as drivers of robotic behavior and self-explanation (refer to Table 1). Inspired by cognitive models like the PSI-theory (Dörner et al., 1999) encompassing the psychological processes of motivation, perception, cognition, and action, researchers equipped robots with simulated physiological and social needs (e.g., energy maintenance, social affiliation) to generate goal-directed actions (Stange and Kopp, 2020; Stange et al., 2022). These needs function as intrinsic motivators, enabling robots to autonomously select strategies, such as seeking charging stations or initiating interactions, while maintaining traceable decision logs for explanation. For example, the Needs Engine architecture allowed robots to articulate behaviors like approaching users with statements such as, “I moved closer because I wanted to interact with you,” linking actions to internal states or robotic intentionality (Arora et al., 2024a). Early transparency mechanisms relied on simplistic outputs, including status lights indicating battery levels or preprogrammed verbal cues like “I need to recharge now” (Stange et al., 2022). However, these systems struggled to contextualize explanations dynamically, often defaulting to generic responses that failed to address users’ situational queries (Matarese et al., 2021).

While innovative, these needs-based models faced criticism for oversimplifying human-like motivation. Robots often exhibited rigid prioritization of needs (e.g., always choosing energy conservation over social interaction), leading to behaviors perceived as antisocial or unpredictable (Alonso and De La Puente, 2018). Additionally, the lack of real-time adaptation in explanation generation limited their utility in complex social scenarios. For instance, a robot might explain its withdrawal from conversation as “I need to charge,” without recognizing the user’s frustration or offering alternative interaction times (Stange et al., 2022). Despite these limitations, this period established critical groundwork for later architectures by demonstrating that robots could generate *self-consistent* behaviors traceable to internal logic that became a precursor to modern interpretable AI systems (Matarese, 2024).

### 3.2 Socio-interactive frameworks

Parallel to needs-based architectures, researchers developed early socio-interactive frameworks to structure robot explanations within human social norms. Building on Malle’s (2003) theory of human attribution, robots were programmed to justify actions using intention-based explanations (e.g., “I opened the door to let you in”) or causal-chain narratives linking perceptions to goals (Matarese et al., 2021) for enhancing user experience (UX) from functional and operational perspectives. For example, the BDI (Belief-Desire-Intention) model (Bratman, 1987) enabled robots to map environmental stimuli (e.g., detecting a user) to desires (social interaction) and subsequent actions (approaching) (Kaptein, 2020). These frameworks emphasized *dialogic interaction*, allowing users to query robots with “Why did you do that?” and receive structured responses tracing decisions to internal states or external triggers.

These early socio-interactive frameworks align with the CASA perspective, suggesting that users subconsciously apply human social rules when interpreting robot behaviors, even when explanations stem from programmed logic. Moreover, Anthropomorphism Theory underscores how attributing human-like intentions to robots influenced early perceptions of trust and transparency, laying a foundation for later UX–CX transitions.

However, early implementations faced three key challenges. First, *explanation granularity* proved difficult to calibrate: overly technical explanations confused users, while overly simplistic ones failed to build trust (Matarese, 2024). Second, real-time processing limitations hindered robots’ ability to generate context-sensitive explanations during fast-paced interactions (Matarese et al., 2021). For instance, a robot might correctly explain its decision to avoid an obstacle but fail to adapt its explanation when the same action inconvenienced a user. Third, users often misinterpreted robots’ intentions due to mismatches between robotic and human social schemas. Studies revealed that users perceived needs-based explanations (e.g., “I was lonely”) as manipulative or anthropomorphic, reducing trust (Stange et al., 2022). These issues highlighted the nascent state of socio-interactive frameworks, underscoring the need for adaptive explanation systems that could balance transparency with social appropriateness (Arora et al., 2021). For example, the initial generation of NAO robots focused largely on enhancing UX, whereas the current iterations of NAO demonstrate a stronger orientation toward CX. By 2018, the field of social robotics had established core principles for explainable social robotics but lacked robust solutions for integrating technical transparency with human-centric communication and social CX behaviors, leading to a gap that would drive innovations in the subsequent transitional period (2019–2022).

## 4 Transitional era: evolution towards user-centered approaches (2019–2022)

### 4.1 Interactive explanation dialog systems

The transitional era (2019–2022) marked a paradigm shift in social robotics, with researchers prioritizing personalized explanation models to address diverse user needs. Building on early needs-based architectures, systems began integrating adaptive dialog flows that adjusted explanation depth based on real-time user feedback. For instance, the VIVA project’s robot Pepper demonstrated how explanation personalization could occur through bidirectional interaction: users could request clarifications like “Why did you move closer?” and receive context-specific responses tracing actions to internal needs (e.g., “I wanted to ensure you heard me clearly”) (Stange et al., 2022). These systems leveraged natural language processing (NLP) to classify user queries into empirically validated explanation types, including contrastive (“Why not approach slower?”) and counterfactual (“What if you stayed still?”) formats (Nchekwube et al., 2023).

Personalization extended beyond verbal exchanges, with robots like NAO employing multimodal strategies combining gestures, gaze, and proxemics to reinforce explanations. Studies showed that users preferred robots that matched explanation complexity to their technical literacy, whereby novices benefited from simplified cause-effect narratives, while experts valued transparency into decision trees. The introduction of adaptive explanation generation frameworks enabled robots to dynamically adjust content based on interaction history, reducing redundancy and improving engagement. However, challenges persisted in balancing personalization with ethical constraints, as over-tailored explanations risked manipulative perceptions when robots overly mirrored user preferences, especially dealing with vulnerable populations (e.g., HRI situations of social robots interacting with students diagnosed with autism) (Arora et al., 2024b).

### 4.2 Impact on user trust and engagement

Concurrent with technical advancements, this era saw the standardization of trust metrics specific to social robotics. The Trust Perception Scale-HRI (TPS-HRI) emerged as a dominant tool, quantifying trust across 14 dimensions like reliability, predictability, and transparency (Schaefer, 2016). Applied to explanation-rich interactions, studies revealed that robots providing need-based explanations (e.g., “I paused because my battery was low”) achieved higher trust scores than those offering purely functional justifications. Early metrics also highlighted cultural variances: collectivist societies prioritized group-aligned explanations (“This helps everyone”), whereas individualist users favored personal benefit narratives. User studies systematically validated explanation effectiveness through mixed-methods approaches. In controlled trials, robots employing interactive elaboration (e.g., answering follow-up questions with incremental detail) reduced user frustration significantly compared to single-turn explanations (Kontogiorgos et al., 2021; Kontogiorgos, 2023), prompting calls for global, cross-cultural validation frameworks (Lim et al., 2021).

Ethical considerations gained prominence as studies revealed unintended consequences of over-trust. Customers reported preferring explainable robots in hospitality and service settings, exhibiting automation complacency, and uncritically accepting erroneous advice from transparent systems (Romeo and Conti, 2025; Ennab and Mcheick, 2024). This paradox highlighted the need for balanced trust calibration, leading to hybrid metrics combining subjective surveys (e.g., TPS-HRI) with behavioral indicators like compliance rates and repair initiation frequency. By 2022, the field converged on a consensus: effective explanations required not just technical accuracy but social resonance, thus aligning robotic logic-based UX functional behaviors with human-normative, CX socio-behavioral expectations. These UX and CX insights set the stage for domain-specific implementations in the subsequent technological maturation phase.

## 5 Maturation era: practical (domain-specific) applications and technological advancements (2023–2025)

### 5.1 Domain-specific implementations

The maturation era witnessed the proliferation of XAI and IAI in sector-specific social robotics applications, driven by advancements in adaptive algorithms and multimodal interaction frameworks. In healthcare, social robots such as, PARO and Pepper demonstrated significant CX improvements by providing real-time explanations of therapeutic actions, reducing patient anxiety, increasing trust (Gongor and Tutsoy, 2025), and improving intentionality and sociality in social robots (Arora et al., 2024a). PARO’s tactile and auditory sensors enabled dementia patients to engage in bidirectional interactions, fostering socio-emotional, CX-based connections through needs-based explanations like “I’m moving closer to hear you better” (De Graaf et al., 2021; Cruz-Sandoval et al., 2024). Retail environments adopted inventory management systems like Walmart’s shelf-scanning robots, which combined computer vision with transparency protocols to explain restocking decisions (Ahmed et al., 2025). These robots improved supply chain efficiency while maintaining user trust through daily operational summaries (Recio-Román et al., 2024; Ahmed et al., 2025). Educational settings integrated NAO and Furhat robots equipped with generative AI to justify pedagogical choices, such as selecting math problems based on student progress (Amirova et al., 2021) or implementing robotic interventions for students with learning disabilities (Arora et al., 2024b). This domain-specific tailoring addressed a critical challenge: aligning explanation modalities with sector-specific expectations, where healthcare prioritized clinical causality and retail emphasized operational efficiency. Additionally, this era marks the emergence of the empowered consumer, with customer experience (CX) becoming increasingly prominent in social robotics and HRI.

### 5.2 Inherently interpretable algorithms

Technological breakthroughs focused on developing algorithms that balanced performance with socio-behavioral transparency. The Large language model and Knowledge graph based Intention Recognition Framework (LKIRF) integrated large language models (LLMs) with knowledge graphs, enabling robots like Pepper to generate step-by-step reasoning traces along with enhancing the intention recognition capabilities of service robots (Zhou et al., 2024). For example, when assisting shoppers, these robots articulated decisions such as “I suggested oatmeal because your purchase history shows gluten sensitivity” (Rajabi and Etminani, 2024; Dehal et al., 2025). Reinforcement learning (RL) architectures incorporated socio-behavioral CX interpretability layers, translating internal states into socially recognizable actions, such as scheduling charging breaks framed as “I need energy to continue helping you” (Akalin and Loutfi, 2021). Hybrid architectures separated core decision-making modules from explanation generators, maintaining task performance and providing granular transparency (Nigro et al., 2025). Standardization efforts and standardized approaches to assessing explainability (XAI) and interpretability (IAI) in social robotics and HRI help achieve technical validity, social alignment, and ethical compliance, paving the way for commercial adoption and advancing the field of explainable and interpretable AI (Gebelli et al., 2025). By 2025, these advancements positioned explainability as a market differentiator, with consumers and users prioritizing transparent and trustworthy robots in service industries and civic settings (Gebelli et al., 2025; Babel et al., 2024).

## 6 Advanced/next-generation era: long-term HRI relationships and future frameworks (2025 – beyond)

Long-Term HRI Relationships 2025 has emerged as a remarkable year that will be leading the advanced/next-generation era. The previous maturation era of social robotics from 2023 to 2025 saw breakthroughs in sustaining human-robot relationships through longitudinal studies and integrated ethical frameworks. Longitudinal trials revealed that robots employing adaptive explanation strategies maintained user engagement with improved trust scores in healthcare and retail settings (Çağıltay, 2025). Personalized interaction models, such as robots that learned user preferences through weekly feedback loops, proved critical for fostering emotional bonds, particularly among elderly users in assisted living facilities (Breazeal et al., 2019). However, challenges emerged in cross-cultural adaptability and robotic interventions targeting vulnerable populations (Lim et al., 2021; Babel et al., 2024; Arora et al., 2024b; Arora et al., 2022). These findings underscored the necessity of culturally-sensitive XAI architectures to support human-robot partnerships and long-term HRI.

The evolution toward long-term human–robot partnerships also reflect theories from intergroup relations. Intergroup Threat Theory and Speciesism highlight that as robots assume socially embedded roles, users’ emotional comfort and perceived safety depend on mitigating intergroup biases and promoting ethical anthropomorphism (Vanman and Kappas, 2019; Fiestas Lopez Guido et al., 2024).

Commercial integration of interpretable AI systems in 2025 and beyond highlights both opportunities and ethical risks. Platforms like LOVOT and PARO achieved high adoption rates in elderly care by embedding real-time need-based explanations (e.g., “I’m adjusting my volume to hear you better”) (Joshi, 2023). However, over-reliance on emotionally aligned robots raised concerns about anthropomorphism, with users exhibiting unhealthy attachment behaviors (Zhang et al., 2025; Arora et al., 2024a). Regulatory frameworks can address these risks by mandating transparency logs and user-controlled data permissions leading to ethical transparency for human–robot interaction (HRI), trust, and collaboration (Arora et al., 2025a; Arora et al., 2025b).

Future Frameworks Comprehensive frameworks, developed in this era, continue to unify technical, ethical, and functional/social (UX/CX) considerations through three pillars: (1) explanation provenance (tracing robotic decisions to specific training data points); (2) dynamic consent interfaces (allowing users and consumers to adjust explanation granularity); and (3) bias mitigation layers (auditing algorithms for cultural and demographic biases). The EU’s AI Act along with the Machinery Regulation mandated these components for social robots in public sectors, driving standardization and establishing robotics’ safety regulatory framework (Mahler, 2024). The ethical robotics’ frameworks enable robots like Pepper to justify decisions contextually (e.g., “I recommended oatmeal due to your gluten sensitivity”) while maintaining the EU’s GDPR-compliant data practices (Rajabi and Etminani, 2024; Mahler, 2024).

Future directions prioritize resolving tensions between personalization and ethical guardrails. Technical challenges persist in real-time adaptation of explanation modalities during complex group and cross-cultural interactions. Societally, debates continue about robots’ role in shaping human behavior, particularly in education, learning disabilities, and mental health, necessitating ongoing collaboration between roboticists, ethicists, and policymakers (Arora et al., 2024b). As the field evolves, the integration of quantum computing and neurosymbolic AI promises to advance both interpretability and relational depth, potentially redefining human-robot coexistence by 2030.

## 7 Managerial and societal implications

The evolution of explainable and interpretable AI (XAI and IAI) in social robotics offers actionable lessons for managers, designers, and practitioners. Each era summarized in Table 1 illustrates how transparency and interpretability can translate into tangible value for organizations deploying social robots in service, healthcare, education, and retail settings.

For instance, Pepper’s personalized greetings in hospitality and Furhat’s adaptive customer service dialogues demonstrate how XAI-enabled robots can enhance engagement, satisfaction, and operational efficiency. Similarly, NAO’s applications in education and rehabilitation show how interpretable AI supports measurable learning outcomes, while Paro and LOVOT exemplify how emotional transparency builds long-term trust in healthcare and eldercare.

Managers can apply these insights to design service ecosystems that leverage explainable and interpretable robotics not only to automate tasks but also to cultivate human trust, improve brand perception, and sustain customer relationships. Embedding XAI and IAI principles into robot design and CX strategies ensure ethical transparency, mitigates risk, and strengthens organizational readiness for AI-driven transformation.

Beyond technical and experiential outcomes, the integration of interpretable systems into social robots carries significant societal implications. The reviewed studies show that interpretable and self-explanatory robots can strengthen long-term trust and engagement, but also risk over-trust, automation bias, and unhealthy anthropomorphic attachment, particularly in emotionally charged domains such as healthcare, eldercare, and mental health support. These effects are especially salient for vulnerable populations, including older adults, patients with dementia, and students with learning disabilities, for whom explanations can both empower and inadvertently increase dependence on robotic agents (Arora et al., 2024b). Cross-cultural differences in anthropomorphism and perceived threat further complicate how interpretable robots are accepted across societies, underscoring the need for culturally adaptive XAI/IAI designs and trust-calibration mechanisms that avoid manipulative or paternalistic explanation strategies. Emerging regulatory frameworks, such as the EU AI Act, GDPR-aligned data practices, and sectoral safety regulations, respond to these societal challenges by emphasizing transparency logs, user-controlled data permissions, and auditable bias-mitigation processes for socially embedded robots. Taken together, these insights position interpretable social robots not merely as technical artifacts, but as socio-technical actors whose design directly shapes ethical governance, public trust, and the responsible deployment of AI in everyday life.​

## 8 Discussion

This scoping review organized the evolution of explainable and interpretable AI (XAI and IAI) in social robotics into four eras, revealing a clear shift from early rule-based explanations to adaptive and socially grounded interpretability. Early developments centered on transparency mechanisms that enabled users to understand robot actions (Reeves and Nass, 1996), while later eras embraced more dynamic, personalized, and interaction-aware explanation frameworks (Wirtz et al., 2018; Song and Kim, 2022). As the field progressed, XAI/IAI became increasingly tied to user experience (UX) and customer experience (CX), influencing trust, acceptance, and perceived social competence (van Doorn et al., 2017). These findings highlight explainability as both a technical requirement and a socio-behavioral driver of effective human–robot interaction.

Several limitations were evident across the reviewed studies. Many investigations relied on short-term interactions, offering limited insight into how trust and acceptance evolve over extended periods (De Graaf et al., 2021). Cultural influences on robotic acceptance, particularly differences between Western and East Asian contexts, remain underexplored, despite evidence that anthropomorphism and perceived threat vary across cultures (Yogeeswaran et al., 2016; Vanman and Kappas, 2019). In addition, divergent evaluation methods and explanation formats restrict comparability across findings. These limitations reinforce the need for longitudinal studies, culturally adaptive interpretability models, and standardized metrics to assess XAI/IAI effectiveness in social robotics.

To synthesize key gaps identified in the literature, Table 2 summarizes the main areas requiring further exploration across the four eras. Future research should prioritize context-aware and cognitively aligned explanation systems, culturally adaptive XAI/IAI frameworks, long-term evaluations of trust and CX, and cross-domain scalability of hybrid interpretability models. As socially embedded robots become more prevalent, there is a growing need for transparent governance structures, trust-calibration mechanisms to prevent over-trust (Fiestas Lopez Guido et al., 2024), and exploration of emerging neurosymbolic and quantum-based approaches to explainability. These directions are essential to supporting ethical, transparent, and human-centered social robotics.

**TABLE 2:** Summary of research gaps and future research directions (2015–2025 and beyond).

Era	Key research gaps	Future research directions
Foundational Era (2015–2018)	Explanations were static and rule-based, lacking adaptation. Limited understanding of how transparency affects trust. Minimal integration of socio-cognitive theories into XAI/IAI.	Develop adaptive, context-aware explanation systems. Integrate socio-cognitive frameworks (e.g., CASA, attribution theory). Examine early UX/CX impacts of transparency on trust and acceptance
Transitional Era (2019–2022)	Cultural and demographic variation in acceptance underexplored. Over-reliance on post-hoc XAI rather than interpretable models. Ethical risks of personalization not fully addressed	Build culturally adaptive XAI/IAI models for HRI. Advance inherently interpretable dialog-based architectures. Strengthen ethical guardrails for personalization and trust calibration
Maturation Era (2023–2025)	Sector-specific XAI solutions not generalizable across industries. Limited longitudinal UX/CX evidence for social robots. Few hybrid XAI + IAI systems tested in real-world conditions	Test cross-domain applicability of sector-specific XAI approaches. Conduct long-term UX/CX and trust studies in real deployments. Evaluate hybrid XAI–IAI systems at scale across sectors
Advanced/Next-Generation Era (2025 – Beyond)	Lack of governance frameworks for socially embedded robots. Rising anthropomorphism and over-trust risks. Limited exploration of neurosymbolic or quantum-informed explainability	Develop proactive governance and safety protocols for explainable robots. Create trust-calibration and over-trust mitigation mechanisms. Integrate neurosymbolic/quantum explainability into social-robot decision-making

## 9 Conclusion

This brief research report synthesized a decade of developments in explainable and interpretable AI in social robotics through a four-era framework that reflects the field’s technical, behavioral, and experiential evolution. The findings demonstrate how interpretability has progressed from early rule-based transparency toward adaptive, socially grounded, and ethically aligned explanation approaches that shape UX and CX. The study’s primary contribution lies in integrating theoretical, technical, and practical insights into a unified structure that highlights the central role of explainability in fostering trust, engagement, and effective deployment across healthcare, education, retail, hospitality, and public-service contexts. Looking ahead, advancing culturally adaptive interpretability models, scalable hybrid architectures, transparent governance mechanisms, and long-term evaluation practices will be critical for developing socially intelligent and trustworthy robots capable of meaningful and sustained human interaction.

## Data Availability

The original contributions presented in the study are included in the article/supplementary material, further inquiries can be directed to the corresponding authors.

## Footnotes

1
https://www.thebusinessresearchcompany.com/report/social-robots-global-market-report
